# Supplementary material for: Effect of Phosphorus Precursor, Reduction Temperature, and Support on the Catalytic Properties of Nickel Phosphide Catalysts in Continuous-Flow Reductive Amination of Ethyl Levulinate
Source: Int J Mol Sci. 2022 Jan 20;23(3):1106. doi: 10.3390/ijms23031106 (PMC8835280; doi:10.3390/ijms23031106)
Supplement: Supplementary file 1 [file ijms-23-01106-s001.zip › ijms-1550969-supplementary.pdf]

Supporting information

Effect of phosphorus precursor, reduction temperature, and support on the catalytic properties of nickel phosphide catalysts in continuous-flow reductive amination of ethyl levulinate

Yazhou Wang [1], Alexey L. Nuzhdin [2,*], Ivan V. Shamanaev [2], Evgeny G. Kodenev [2], Evgeny Yu. Gerasimov [2], Marina V. Bukhtiyarova [2] and Galina A. Bukhtiyarova [2]

[1] Novosibirsk State University, Novosibirsk, 630090, Russia

[2] Boreskov Institute of Catalysis SB RAS, Novosibirsk, 630090, Russia

E-mail: anuzhdin@catalysis.ru

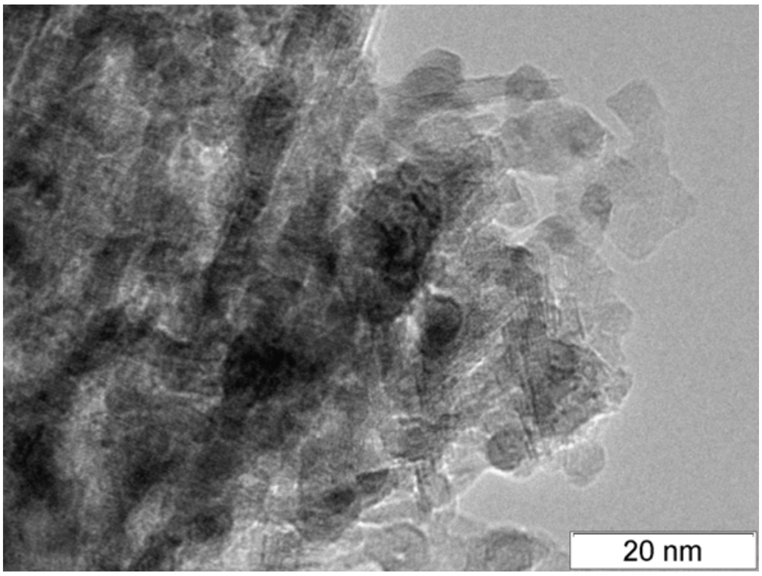

(a)

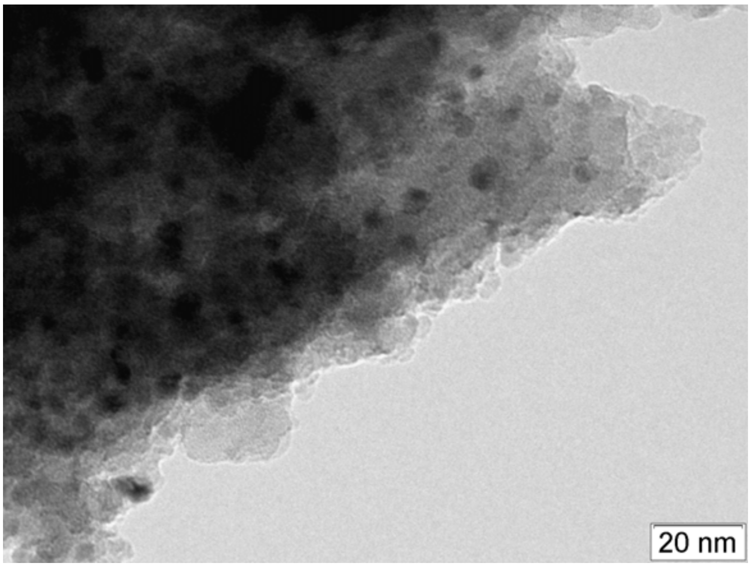

(b)

Figure S1 TEM data of (**a**) Ni/Al_2O_3 and (**b**) Ni/SiO_2.

Table S1. Physicochemical properties of the supports and diluters.

Catalyst	S_{BET}, m^2 g^{-1}	V_{pore}, cm^3 g^{-1}	NH_3-TPD, μmol g^{-1}
SiO_2	300	0.80	84
γ-Al_2O_3	235	0.79	421
SiC	1	–	0
SAPO-11	295	0.26	1110
zeolite β	609	0.49	1920
